# Predicting Adherence to Computer-Based Cognitive Training Programs Among Older Adults: Study of Domain Adaptation and Deep Learning

**DOI:** 10.2196/53793

**Published:** 2024-09-16

**Authors:** Ankita Singh, Shayok Chakraborty, Zhe He, Yuanying Pang, Shenghao Zhang, Ronast Subedi, Mia Liza Lustria, Neil Charness, Walter Boot

**Affiliations:** 1 Department of Computer Science, Florida State University, Tallahassee, FL, United States; 2 School of Information, Florida State University, Tallahassee, FL, United States; 3 College of Medicine, Florida State University, Tallahassee, FL, United States; 4 Division of Geriatrics and Palliative Medicine, Weill Cornell Medicine, New York, NY, United States; 5 Department of Psychology, Florida State University, Tallahassee, FL, United States

**Keywords:** domain adaptation, adherence, cognitive training, deep neural networks, early detection of cognitive decline

## Abstract

**Background:**

Cognitive impairment and dementia pose a significant challenge to the aging population, impacting the well-being, quality of life, and autonomy of affected individuals. As the population ages, this will place enormous strain on health care and economic systems. While computerized cognitive training programs have demonstrated some promise in addressing cognitive decline, adherence to these interventions can be challenging.

**Objective:**

The objective of this study is to improve the accuracy of predicting adherence lapses to ultimately develop tailored adherence support systems to promote engagement with cognitive training among older adults.

**Methods:**

Data from 2 previously conducted cognitive training intervention studies were used to forecast adherence levels among older participants. Deep convolutional neural networks were used to leverage their feature learning capabilities and predict adherence patterns based on past behavior. Domain adaptation (DA) was used to address the challenge of limited training data for each participant, by using data from other participants with similar playing patterns. Time series data were converted into image format using Gramian angular fields, to facilitate clustering of participants during DA. To the best of our knowledge, this is the first effort to use DA techniques to predict older adults’ daily adherence to cognitive training programs.

**Results:**

Our results demonstrated the promise and potential of deep neural networks and DA for predicting adherence lapses. In all 3 studies, using 2 independent datasets, DA consistently produced the best accuracy values.

**Conclusions:**

Our findings highlight that deep learning and DA techniques can aid in the development of adherence support systems for computerized cognitive training, as well as for other interventions aimed at improving health, cognition, and well-being. These techniques can improve engagement and maximize the benefits of such interventions, ultimately enhancing the quality of life of individuals at risk for cognitive impairments. This research informs the development of more effective interventions, benefiting individuals and society by improving conditions associated with aging.

## Introduction

### Background

Cognitive decline in the aging population presents an unprecedented challenge for the United States and the world. The World Health Organization predicts that the global population of those 60 years and older will double from 1.4 billion in 2020 to 2.1 billion in 2050 and will triple for those 80 years and older reaching 426 million [1]. While some cognitive changes are normal with aging, impacting the performance of some everyday tasks, individuals experiencing nonnormative cognitive declines face even greater challenges. The risk of developing dementia and Alzheimer disease is substantial, with dementia being the seventh leading cause of death worldwide [1]. As the population continues to age, and despite declines in dementia prevalence [2] the number of people affected by dementia is projected to triple by 2050, along with the associated costs [3].

Addressing age-related cognitive changes is crucial for individuals’ well-being and independence, and has broader societal impact. Cognitive training interventions, such as technology-based exercises targeting specific cognitive functions, have been widely used. However, research on their effectiveness has yielded both positive [4-6] and negative [7-9] results. Adherence to these training programs is essential for understanding and maximizing their benefits, but factors influencing adherence are not fully understood [10,11]. The ongoing Adherence Promotion with Person-Centered Technology project aims to understand adherence barriers, develop predictive algorithms, and support early detection of age-related cognitive decline. By predicting adherence, personalized reminders can be sent to encourage participants to adhere to the prescribed training schedule.

Our work differs from previous related research in 2 fundamental ways. Previous research efforts mostly used personality traits and metacognitive beliefs of each participant for predicting their adherence to training regimes [12-15]. These are static “distal” features, which quantify the personality and attitudes of a participant, and are unlikely to bear as much information about their continuing engagement to the training program as more proximal dynamic features such as aspects of current adherence behavior. Advances in computing technologies and computational approaches allow us to tailor interventions based on more objective, automatically collected data rather than on mostly subjective, self-reported measures, as well as to create highly adaptive, dynamic systems that can support different types of behavior change interventions [16,17]. We used relevant information about the engagement of a participant to the training schedule (eg, engagement time, number of tasks performed) for N consecutive days to predict their adherence for the (N+1)th day. These predictors contain richer and fine-grained contextual information about the participant’s engagement and may thus be much more informative in predicting adherence. Further, while previous research mostly used naïve regression models for predicting the adherence of a participant [12-15], we used sophisticated deep learning models for the same purpose.

In our recent research, we demonstrated the potential of deep convolutional neural networks (CNNs) to predict adherence for a particular participant based on their past behavior, using data from previous computer-based cognitive intervention studies [18,19]. To capture the individualistic differences in participation, we developed a separate prediction model for each participant, rather than a single model for all participants. While our empirical results were promising, one challenge we faced was the limited amount of labeled training data per participant (30 days only) to train a reliable deep neural network. Our main objective in this research was to address this practical challenge by using relevant training data from other participants to train a prediction model for a given participant. This is a challenging task, as each participant has a unique pattern of engagement with the training schedule, and directly using the training data from other participants, without addressing the individualistic variabilities, may not produce the optimal model for a given participant.

Domain adaptation (DA) techniques are instrumental in leveraging ample training data from source domains to train a reliable model for a related target domain of interest, where labeled data are scarce, when the data of each domain are derived from a different probability distribution [20,21]. Through DA, the disparity between the source and target domains can be minimized, so that the training data from the source domains can also be used to train a model for the target domain, thereby addressing the challenge of limited labels. We used state-of-the-art adversarial DA techniques [22], together with advanced signal processing algorithms to overcome the challenge of limited training data for each participant.

Our study highlights the potential of using deep neural networks and DA techniques to predict adherence lapses and develop personalized support systems for cognitive training interventions. Such a prediction system can improve engagement and help maximize the benefits of such interventions, ultimately enhancing the quality of life for individuals with cognitive impairments. To the best of our knowledge, this research is the first of its kind to use advanced deep DA techniques to predict older adults’ daily adherence to cognitive training programs.

### Objectives

This study sought to (1) explore the potential of deep learning to predict a participant’s adherence to cognitive training programs, based on their past behavior; (2) investigate if applying DA techniques can help improve the accuracy of predicting adherence for a given participant, by using relevant information from other participants in the same or different clinical trial; and (3) determine if time series data can be presented in a more meaningful and understandable way, and if it can be used to cluster the participants based on their adherence to a cognitive training program.

## Methods

### Research Design

We used the Mind Frontiers (Aptima Inc) cognitive training software package in this study. The video game application included 7 Wild West–themed mini-video games. These games were designed to improve memory, attention, spatial processing, task-switching, reasoning ability, and problem-solving, and were played on a Lenovo 10 tablet. Participants were trained on how to use the tablet and play each game; after each game, participants received feedback and the difficulty of the game was adjusted based on their previous performance.

### Ethical Considerations

Florida State University Institutional Review Board approved this study’s protocol (2017.20622) and informed consent form. Informed consent was obtained from all participants, and participants were given the opportunity to opt out at any time. This consent process included instituional review board–approved language that collected data could be made available for secondary analysis by other research teams and that any shared data would not include personal identifiers. Data were labeled with participant IDs, meaning that data were deidentified in this study. Participants were compensated a total of US $200 (study 1) or US $75 (study 2) for their participation, with the difference driven by the shorter commitment involved in study 2.

### Datasets

In total, 2 datasets were used in this study, which we will refer to as the study 1 and study 2 datasets. Study 1 data [13] came from a cognitive training study that involved 2 phases. In phase 1, participants were asked to follow a prescribed schedule for 12 weeks, playing for 5 days each week, for at least 45 minutes per day. Phase 2 was unstructured, where participants were encouraged to play as frequently as they wanted to, for 6 weeks. In this study, only the data collected during the structured phase was analyzed, as adherence cannot be defined without the proposed game-playing instructions given in phase 1. This study had 118 participants, with an overall mean age of 72.6 years and an SD of 5.5 years. Further, 78 (66.2%) participants were female and 40 (33.8%) were male. The cognitive training program consisted of 7 different tasks with 5 possible outcomes (defeat, stalemate, victory, abandonment, or not yet completed). Participants were also given a custom user manual on how to operate the tablet and play each game within the intervention and were provided information on how to access technical support.

The study 2 dataset came from a similar but independent study [12]. Here, in phase 1, participants were asked to follow a prescribed schedule for 8 weeks, playing for 5 days each week, for at least 60 minutes per day. Phase 2 was unstructured and lasted for 4 weeks. A total of 120 adults aged 64 years and older were recruited from Leon County, Florida, and the surrounding area for this study. Among the participants, 116 completed the structured phase of this study. The average age of the participants was 72.6 (SD 5.5) years, with a range of 64‐84 years. In total, 77 (64.2%) participants were female and 43 (35.8%) were male. The intervention also involved use of the tablet-based Mind Frontiers app (version 2.4.11), which included 7 gamified neuropsychological tasks targeting working memory, processing speed, executive control, and spatial reasoning. While the intent was for participants to complete both phases of this study, the second phase of this study did not require participants to engage with the gaming intervention for any predetermined length of time. Therefore, we only used the data from the first phase of this study. To increase the comparability of datasets, all analyses focus on the first 8 weeks of intervention engagement. Note that variations in study parameters make the datasets slightly different (eg, 45 min vs 60 min sessions).

### Deep Neural Networks for Adherence Prediction

We used deep CNNs to predict adherence from the multivariate time series data. While CNNs are well-suited for image recognition tasks, they have also been successfully used for time series data classification [23-25]. The primary mechanism used by these models is convolution, which allows the network to extract essential features from the raw data. In 1D convolution, the kernel slides unidirectionally from the start of the time series to its end. If we have input vector *f* of length *n* and a kernel *g* of length *m*, the convolution *f × g* of *f* and *g* is defined as follows:


(1) $$\left(f\times g\right)\left(i\right)=\sum_{j=1}^{m}g\left(j\right)\cdot f\left(i-j+\frac{m}{2}\right)$$

**Figure 1.**
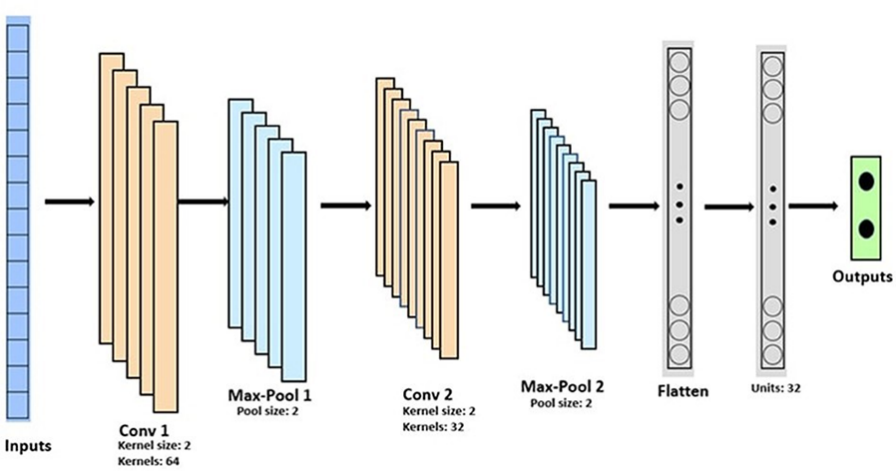
The convolution neural architecture used in this study (adapted from Singh et al [19], which is published under Creative Commons Attribution 4.0 International License [26]). Conv: convolution.

In this research, we used 2 convolution blocks consisting of a convolution layer and a maximum pooling layer for feature extraction. The maximum pooling layer moves a pool of predetermined size across the input and calculates the maximum of the region. These blocks are followed by a dense and an output layer. Figure 1 shows the architecture of the CNN used in this research.

**Figure 2.**
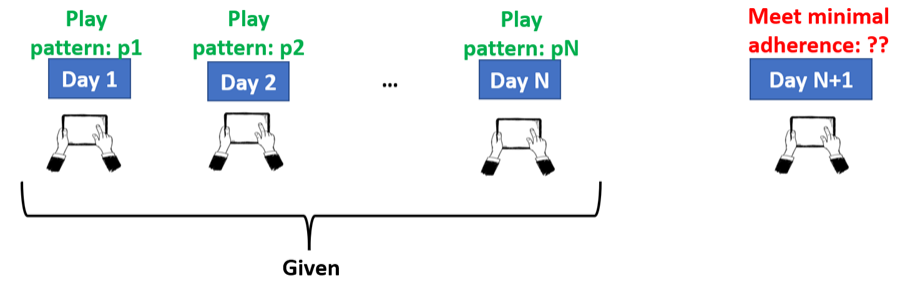
Given the play patterns of a participant for N consecutive days, our goal was to predict whether the participant will meet the minimal adherence criterion on the (N*+*1)th day (adapted from Singh et al [19], which is published under Creative Commons Attribution 4.0 International License [26]).

Following the setup in our previous research [18,19], we used the first 30 days of data for each participant for training and the next 30 days for testing. We used 4 time-dependent variables obtained through game interface interactions as our predictors, namely, (1) duration for which a participant played, (2) number of sessions, (3) maximum level reached, and (4) the number of tasks performed. Session refers to daily session, the number of days on which gameplay occurred. Participants were asked to play 5 sessions (on different days) each week. A session, however, was comprised of the completion of a number of tasks (or games). The participants were said to be satisfying the minimal adherence criterion if they played for at least 10 minutes on a given day. This minimal adherence criterion was selected to ensure that the participants spent a reasonable amount of time playing the game and to avoid cases where they may have accidentally opened the app and then backed out. Hence, this was considered as the threshold to define the 2 classes (adherent and nonadherent) in our study. Based on the values of these predictors for a given participant for N days, our goal was to predict the adherence class of the participant for the (N*+*1)th day, N being the window size. This is depicted in Figure 2. Note that participants were asked to play on 5 out of 7 days per week. As such, nonengagement was permitted 2 days per week, and a participant could still be classified as adherent overall for the week by not playing for 2 days. The ability to predict whether participants engage or not on a specific day, however, is crucial for facilitating the early detection of adherence lapses so that our system under development can provide just-in-time adherence support. In this paper, we focus on the artificial intelligence aspect of the problem, predicting whether a participant will adhere to the training schedule on any given day, given their past N days of data (as shown in Figure 2). A separate, personalized deep neural network was trained for each participant to capture the individual playing patterns, rather than attempting to fit a single model for all participants.

### DA for Adherence Prediction

One of the main challenges in our problem setup was the limited amount of labeled data per participant (30 d) to train a reliable deep neural network. DA or transfer learning is a technique used in machine learning to address the challenge of model training with limited labeled data. The fundamental premise of DA is to leverage abundant labeled training data in one or more source domains to train a model for a target domain of interest, where labeled data are scarce [20-22]. The source and the target data are derived from different probability distributions and thus a model trained on the source domains may not generalize directly to the target domain. This necessitates a strategy to address the disparity between the probability distributions of the source and target domains so that the labeled source samples can be used to train a robust model for the target domain. While extensively used in computer vision, DA has also been successfully used with time series data [27,28].

DA techniques based on adversarial training have depicted promising empirical performance. We used the domain adversarial network in our study (please refer to [22] for further details). The architecture includes a deep feature extractor, together with 2 classification heads: one to classify the label y of a given sample (adherent or nonadherent in our case) and the other to classify the domain d from which a given sample is derived (source or target). During model training, the label classifier was trained to achieve high accuracy (so that the test samples are accurately classified), but the domain classifier was trained to achieve minimal accuracy. This ensures that the deep model learns domain invariant features and a classifier trained to distinguish between the source and target domain samples has high error. This also ensures that the disparity between the 2 domains is addressed, and the labeled source samples can be used to train a model for the target domain. The conventional cross-entropy loss is used to train the label classifier; the domain classifier is connected to the feature extractor via a gradient reversal layer that multiplies the gradient by a certain negative constant during the backpropagation-based training so that the domain classifier is trained to have a high error. Please refer to Ganin et al [22] for more details about this method.

In our problem setup, a particular participant constitutes the target domain and each of the other participants constitutes the source domain. Each participant had a unique playing pattern, which implies that the data for each participant is derived from a different probability distribution. Our objective was to use data from the source participants to augment the training data for the target participant and to train a better adherence prediction model for the target participant. To deal with the scale of the data, and to ensure that the source participants had similar playing patterns as the target participant (so that only relevant information is transferred), we exploited a clustering technique based on Gramian angular fields (GAFs), as described below. We clustered the participants based on their playing patterns and the DA scheme was applied cluster-wise; that is, a particular participant was considered as the target, and all the other participants in the same cluster (instead of the whole dataset) were considered as the source participants.

### Clustering Time Series Data Using GAFs

Directly clustering temporal cognitive training data can be challenging due to its complex and dynamic nature. We hence used GAFs to transform the univariate time series data (time spent in playing games) into images [29-31]. GAF images provide a more intuitive representation of the data, making it easier to interpret and analyze. As mentioned before, the adherence classes were defined based on how long each participant engaged in the training schedule on a particular day. We therefore used the variable “time spent in playing games” to transform the (univariate) time series data into images and cluster them, instead of the other variables: “number of sessions,” “maximum level reached,” and “number of tasks performed.” This ensured that participants with similar adherence patterns belonged to the same cluster; thus, during DA, only participants with similar playing patterns as the target participant can be used to transfer relevant knowledge.

**Figure 3.**
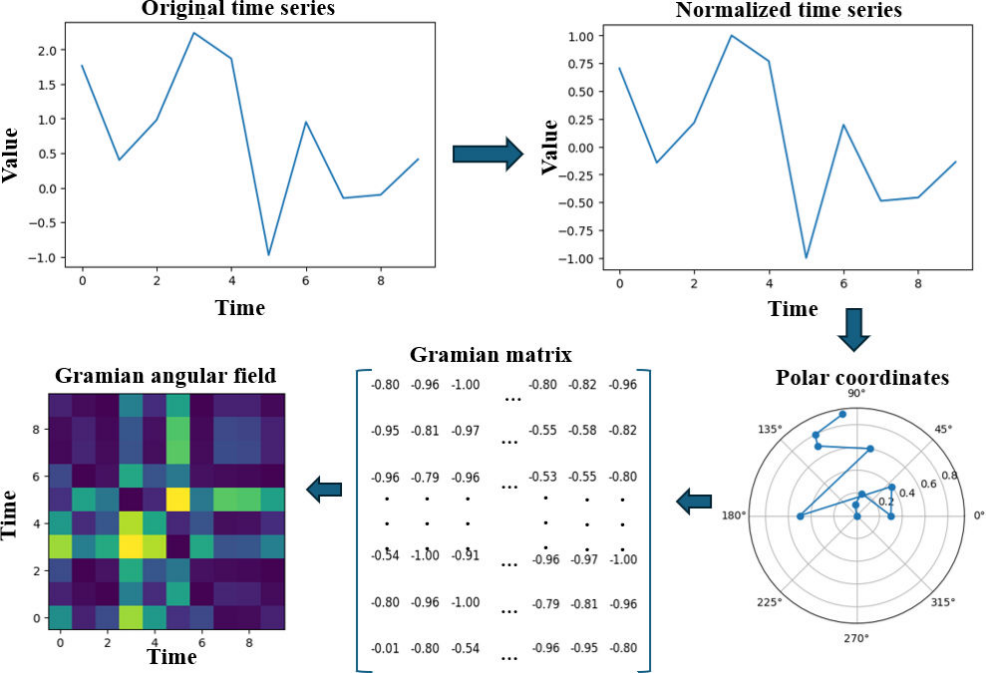
Pipeline for converting time series data into GAF images. GAF: Gramian angular field.

The process of converting time series to GAFs involves several steps, as detailed below (the complete pipeline is depicted in Figure 3):

- Normalizing the time series data: The time series data are first normalized to have a mean of 0 and an SD of 1. This step is important to ensure that the resulting GAF images capture the underlying patterns and trends in the data.

- Conversion to polar coordinates: The time series data were then mapped to the polar coordinate system where each point on the plane is determined by its distance from a reference point and an angle from a reference direction. Assuming our time series is composed of *T* timestamps with corresponding values *x_i_*, then the angles are computed as *arccos(x_i_).* They lie within [0, π]. The radius is computed using time stamp *i*, where *i*∈ *T* represents the timestamp of data point *x_i_*.

Mathematically it translates to,


(2) $$\left\{ \begin{array}{l} \theta_{i}=across(x_{i}),\;\;-1\leq x_{i}\leq 1\\ r=i/T \end{array}\right.$$

- Generating a Gramian matrix: The normalized and converted time series data were then used to generate a Gramian matrix using a chosen mathematical function, such as the cosine or sine function. The Gramian matrix captures the pairwise inner products between the time series data points, which provides a measure of the similarity between different points in the time series.5

(3) $$GAF=\left[\begin{array}{ccc} \cos\left(\theta 1\;+\;\theta 1\right) & \ldots & \cos\left(\theta T\;+\;\theta 1\right)\\ \cos\left(\theta 1\;+\;\theta 2\right) & \ldots & \cos\left(\theta T\;+\;\theta 2\right)\\ \vdots & \ddots & \vdots \\ \cos\left(\theta 1\;+\;\theta T\right) & \ldots & \cos\left(\theta T\;+\;\theta T\right) \end{array}\right]$$

- Mapping the Gramian matrix to an image: The Gramian matrix was then mapped to a 2D image using a color mapping scheme, such as a grayscale or rainbow colormap as shown in Figure 3. The resulting image captures the underlying patterns and trends in the time series data.

### Clustering of Images

**Figure 4.**
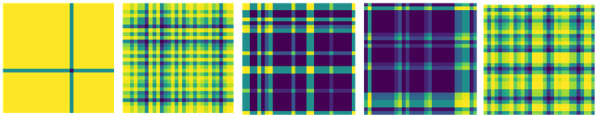
Examples of generated GAF images. GAF: Gramian angular field.

We converted the univariate time series data of the length of playtime for each participant into GAF images using the cosine function, to capture the underlying patterns in cognitive training performance [32,33]. We then used the pretrained Visual Geometry Group 16 deep neural network to extract features from the GAF images, which were fed into the K-means clustering algorithm to group the participants into distinct clusters based on their daily playing time. Sample images from different clusters are shown in Figure 4. The GAFs are symmetrical along the diagonal, and the colors vary from yellow to blue with green gradients in between. The yellow or lighter color means that the participant did not play or spent much less time playing, while the increase in the gradient from yellow to blue implies that the participant spent more time playing. The first image in Figure 4 comes from a participant who did not play at all (GAF image is mostly yellow). The third and fourth images come from participants who played more consistently; however, there were some days in between where they either did not play or played for a short duration, which explains the yellow regions in between. The second and fifth images come from participants who were erratic in their playing patterns.

The advantages of converting the time series data into images and clustering them were twofold: (1) the resulting clusters allowed for a more visually interpretable understanding of the data and enabled us to identify distinct trends in the playing patterns of the participants, which can be used to tailor cognitive training programs to specific groups of individuals; and (2) it enabled us to identify the relevant source participants (who have similar playing patterns) for a given target participant, while applying DA to train a model for the target participant.

### Experimental Setup

For each participant, we used the first 30 days of data for training and the next 30 days for testing. This split was selected to strike a balance between the training and testing samples. Further, there is evidence in the psychology literature showing that the median time to develop different health-related habits asymptotes around 30 days [34]. The proposed split can thus be useful to assess long-term adherence. The input to the CNN was the multivariate time series data consisting of the maximum task level reached, number of sessions played, time spent playing, and number of tasks completed each day. A threshold of 10 minutes on the play time on a particular day was used to determine the classes (adherent or nonadherent); that is, if a participant played for more than 10 minutes on a given day, they were considered to adhere to the training program on that day; otherwise, they were considered to be nonadherent to the program on that day. Given the playing data of a particular participant over N days, our goal was to train deep neural networks to predict the adherence class on the (N*+*1)th day. As detailed in our previous research [19], we used the fast Fourier transform to detect the presence of any seasonality in the time series data. Fast Fourier transform was applied to transform the time series training data into the frequency domain and derive the corresponding amplitude and frequency. The highest amplitude frequencies represent seasonal patterns and the lowest amplitude frequencies represent noise. The inverse fast Fourier transform was then applied to the frequency with maximum amplitude to obtain the time interval for the most prominent cycle (periodic pattern). This cyclic period was used as the window size N*,* which was computed as *7* in our study. A separate deep model was trained for each participant to capture individualistic playing patterns. After every epoch, the error of the model on the validation set was noted to avoid overfitting.

The data from a particular clinical trial were first clustered using GAFs. We experimented with different numbers of clusters and manually inspected the GAFs within each cluster. With 4 clusters, the GAFs within each cluster visually appeared to be similar, denoting that participants with similar playing patterns were grouped in each cluster; with other values of *k*, each cluster had mixed GAFs, denoting that each cluster had participants with varied playing patterns. We therefore performed K-means clustering with (K=4) clusters in our empirical studies. Further, 1 participant from a given cluster was selected as the target participant, and all the other participants in the same cluster were designated as the source participants. A deep model was trained for the target participant and was evaluated on the test data for the same participant. The process was repeated for all participants across all the clusters and the final score was computed as the average score over all the participants. In total, 4 evaluation metrics were used: precision, recall, *F*_1_-score, and accuracy. Precision, recall [35], and *F*_1_-score [36] were used to measure the model’s performance on positive predictions, while accuracy was used to evaluate the overall model performance. We conducted 3 different sets of experiments:

1. No source, no DA: In this setup, only the 30 days of training data for the target participant was used to train a model for that participant; the data from the other participants was not used at all.
2. With source, no DA: here, in addition to the training data of the target participant, the training data from the source participants (in the same cluster as the target participant) was also used to train a model for the target participant. However, the training data from the source participants was used directly, without applying any DA.
3. With source, with DA: In this setup, DA was used to address the domain disparity between the target and source participants, and the training data from the source participants (in the same cluster) was used to train a model for the target participant.

In all 3 setups, the trained model was tested on the test data of the target participant. For the experiments without DA, the CNN architecture in Figure 1 was used, while for experiments with DA, the layers before the flatten layer were used as the feature extractor as in .

## Results

### Experiments With a Single Dataset

The results on the study 1 and study 2 datasets are depicted in Tables1  2, respectively. Note that all the evaluation metrics referred to in this section are the average values of all the participants across all clusters. For the study 1 dataset (Table 1), using training data only from the target participant, without any source or DA, resulted in an accuracy of 63% (the accuracy was computed as the percentage of times our model correctly predicted whether a given participant will meet the minimal adherence criterion on day N+1, as illustrated in Figure 2; this was averaged across all the participants). Using training data from the other participants (source) in the same cluster as the target participant, but without DA, resulted in an improved accuracy of 67.9%. Using training data from the source participants together with DA produced the best accuracy of 71.7%. The superior performance of DA is also reflected in all the other evaluation metrics. The same pattern is evident for the study 2 dataset (Table 2), where using the training data from the source participants (in the same cluster as the target participants) together with DA consistently furnished the best performance. Notably, using DA, both the accuracy and *F*_1_-score improved by more than 8% compared to the “no source, no DA” experiment.

We also note that the “with source, no DA” experiment produced slightly better results than the “no source, no DA” experiment. This shows that using training data from the source participants can improve the adherence prediction performance; however, using the data directly without addressing the domain disparity may not produce optimal results. The best results were obtained when the training data from the source participants were used, and the difference in probability distribution between the source and the target participants was addressed through DA.

We also conducted statistical tests of significance on the *F*_1_-score using the ANOVA repeated measures (the *F*_1_-score may be a better indicator of the overall performance than accuracy, to address the presence of any class imbalance in the data). We compared the performance of the CNN models for the following two experimental setups: (1) with source, no DA, and (2) with source, with DA. A statistically significant difference (*P*=.004) was found between the results of using DA compared to not using DA for the study 2 dataset. A sign test was also conducted on the *F*_1_-score of the individual participants to test for consistent differences in the results with and without using DA. A significant difference (*P*=.003) was found in the results for the study 2 dataset. These results corroborate the potential of DA algorithms to develop personalized deep learning models for predicting adherence to cognitive training programs in older adults.

**Table 1.** Results (in percentage) on the study 1 dataset. Italicized values represent the best performance. The scores are calculated by averaging the respective scores over all the participants.

Experiment type	Accuracy, mean (SD)	Precision, mean (SD)	Recall, mean (SD)	*F*_1_-score (SD)
No source, no DA^a^	63 (0.2)	58.3 (0.2)	57.1 (0.2)	54.3 (0.3)
With source, no DA	67.9 (0.3)	61.1 (0.3)	60.2 (0.3)	58.7 (0.3)
With source, with DA	*71.7 (0.3)*	*63.2 (0.3)*	*62.3 (0.3)*	*59.4 (0.3)*

a DA: domain adaptation.

**Table 2.** Results (in percentage) on the study 2 dataset. Italicized values represent the best performance. The scores are calculated by averaging the respective scores over all the participants.

Experiment type	Accuracy, mean (SD)	Precision, mean (SD)	Recall, mean (SD)	*F*_1_-score (SD)
No source, no DA^a^	57.3 (0.2)	54.1 (0.2)	52.4 (0.2)	49.6 (0.2)
With source, no DA	61.8 (0.2)	54.5 (0.2)	53.4 (0.2)	50.1 (0.2)
With source, with DA	*66.6 (0.2)*	*60 (0.2)*	*60.4 (0.2)*	*57.6 (0.2)*

a DA: domain adaptation.

### Experiments With a Combination of the Two Datasets

Having validated the usefulness of DA on single trial datasets, we conducted an experiment to study whether data from participants from a different clinical trial can also help in training a predictive model for a target participant in a given clinical trial. To this end, we segregated all the participants in both datasets together, into 4 clusters using the GAF method. Within each cluster, for a given target participant, only participants from the other dataset (different from the target participant’s dataset) were considered as source participants. This was accomplished to mimic the following challenging real-world scenario: a participant enrolls in a cognitive intervention program, and we desire to train a deep neural network to predict adherence for this participant; we have access to a limited amount of training data from this participant. However, we do not have any training data available from other participants in the same program; rather, we have access to data from other participants from a similar (previously conducted) intervention program. Our goal, therefore, was to leverage data from a similar intervention program, in addition to the limited labeled data for the target participant, to develop an adherence prediction model for the target participant. Note that, the only objective of clustering the participants in both datasets together, was to identify the source participants for a given target participant. Once the source participants were identified, the prediction model was trained for the target participant using data from the source participants; no prediction model was trained on the combination of the 2 datasets. The same 3 experiments were conducted, and the results are presented in Table 3.

The results depict a similar pattern, with DA comprehensively outperforming the other 2 experiments. The *F*_1_-score increased by almost 8% compared to the scenario where the source data are used directly without applying DA. As before, a statistical test of significance was conducted on the *F*_1_-score using the ANOVA repeated measures. A significant trend (*P*=.08) was found between the results of using DA compared to using the source data directly for training without DA. A sign test was also conducted on the *F*_1_-score of the individual participants; a significant difference (*P*=.004) was found in the results with and without using DA. We also conducted a sign test on the combined results obtained from the 3 experiments (study 1, study 2, and study 1 and study 2 combined). A significant difference (*P*<.001) was found in the results of the individual participants with and without using DA. These findings suggest that incorporating other participants’ data by using DA techniques can improve the generalization capability of a deep neural network trained to predict adherence of a particular participant, even when the other participants’ data are derived from a different (but similar) intervention program.

Overall, the findings of this study suggest that DA has tremendous promise in improving the performance of a deep neural network trained to predict adherence to a cognitive training program of a given participant, by appropriately leveraging training data from other participants. Such a framework can be immensely useful in situations where only a limited amount of training data is available for the target participant. DA consistently produced better performance compared to baseline methods which did not incorporate data from other participants (source participants) at all or used the data directly without DA. Additionally, ANOVA repeated measures analyses demonstrated a statistical trend toward significance in the *F*_1_-score between experiments with and without DA while using the source data, further corroborating the efficacy of DA on adherence prediction accuracy.

**Table 3.** Results (in percentage) on study 1 and study 2 datasets. In this experiment, for each cluster, a participant is selected as the target and all the participants, from the other dataset, in the same cluster are considered as source. Italicized values represent the best performance. The scores are calculated by averaging the respective scores over all the participants.

Experiment type	Accuracy, mean (SD)	Precision, mean (SD)	Recall, mean (SD)	*F*_1_-score (SD)
No source, no DA^a^	64.2 (0.3)	57.1 (0.3)	58.2 (0.2)	54.2 (0.3)
With source, no DA	66.9 (0.3)	51.3 (0.3)	57.3 (0.2)	50.9 (0.3)
With source, with DA	*68.5 (0.2)*	*61.3 (0.2)*	*60.4 (0.2)*	*58.7 (0.2)*

a DA: domain adaptation.

## Discussion

### Principal Results

The results of this study have several important implications for the field of adherence prediction in the context of gamified cognitive intervention programs. Our findings suggest that CNNs can be a powerful tool for analyzing time series data and predicting adherence and can have important implications for the field of cognitive neuroscience. By using CNNs to predict adherence, we were able to identify patterns and trends in the data that could be used to improve cognitive training interventions, ultimately leading to enhanced quality of life for individuals with cognitive impairments. This research has implications for the development of more effective intervention approaches that can benefit individuals and society by improving conditions associated with aging and the life course.

More importantly, each participant has a unique playing pattern, and it is important to address the individualistic variabilities when developing adherence prediction models for cognitive gaming programs. Our study demonstrated that DA techniques can effectively reduce domain shift and improve the accuracy of CNN models in predicting adherence. The results of the experiments conducted on the study 1 and study 2 datasets demonstrated the effectiveness of DA in improving adherence prediction performance. This suggests that appropriately leveraging information from participants with similar playing patterns can enhance the accuracy of adherence pattern predictions. To the best of our knowledge, this is the first research effort to study the performance of advanced deep DA techniques to predict adherence to cognitive training programs for older adults.

Our study also suggests that GAFs can be a valuable tool for preprocessing time series data and clustering participants based on their playing patterns. The findings indicate that GAFs can aid in identifying participants with similar behaviors and interpret the patterns visually. They also enable us to identify source participants that have similar playing patterns as the target participant, so that relevant information can be transferred during DA.

### Conclusions

In conclusion, this study highlights the potential of deep neural networks and DA techniques in predicting adherence lapses and developing personalized support systems for cognitive training interventions. The findings contribute to the advancement of the field and provide valuable insights for improving engagement and maximizing the benefits of such interventions for individuals with cognitive impairments. Future research will further explore the application of these techniques in other contexts and populations with the fundamental goal of improving the effectiveness of cognitive training interventions.

## Abbreviations

CNN: convolutional neural network
DA: domain adaptation
GAF: Gramian angular field
